# Ether and Chloroform

**Published:** 1874-11

**Authors:** 


*MEDICAL EXAMINER—October*, 1874:

PROF. SCHIFF ON THE DIFFERENCE IN THE ANÆSTHESIA PRODUCED BY CHLOROFORM AND ETHER.—As a result of several thousand experiments on animals, the author has come to the conclusion that ether *gradually* suspends the processes of respiration and circulation, so that it is in the power of the operator to save the animal at any given moment, while the suspension produced by chloroform is not gradual but irregular, the circulation often ceasing while respiration still exists. The author would, therefore, decide *that the physician is responsible for any death by ether, but not for a fatal issue produced by chloroform.*

